# Derivation of a Germline Competent Transgenic Fischer 344 Embryonic Stem Cell Line

**DOI:** 10.1371/journal.pone.0056518

**Published:** 2013-02-20

**Authors:** Hongsheng Men, Elizabeth C. Bryda

**Affiliations:** Rat Resource and Research Center, Department of Veterinary Pathobiology, University of Missouri, Columbia, Missouri, United States of America; Universidade de São Paulo, Brazil

## Abstract

Embryonic stem (ES) cell-based gene manipulation is an effective method for the generation of mutant animal models in mice and rats. Availability of germline-competent ES cell lines from inbred rat strains would allow for creation of new genetically modified models in the desired genetic background. Fischer344 (F344) males carrying an enhanced green fluorescence protein (EGFP) transgene were used as the founder animals for the derivation of ES cell lines. After establishment of ES cell lines, rigorous quality control testing that included assessment of pluripotency factor expression, karyotype analysis, and pathogen/sterility testing was conducted in selected ES cell lines. One male ES cell line, F344-Tg.EC4011, was further evaluated for germline competence by injection into Dark Agouti (DA) X Sprague Dawley (SD) blastocysts. Resulting chimeric animals were bred with wild-type SD mates and germline transmissibility of the ES cell line was confirmed by identification of pups carrying the ES cell line-derived EGFP transgene. This is the first report of a germline competent F344 ES cell line. The availability of a new germline competent ES cell line with a stable fluorescence reporter from an inbred transgenic rat strain provides an important new resource for genetic manipulations to create new rat models.

## Introduction

Because rats share similarities in their anatomy and physiology with humans, they are often a model animal in biomedical research as well as drug discovery and development. Rats have been widely used in the areas of hypertension, aging, infectious diseases, cancer and neurological disorders [1]. Besides physiological similarities, the larger size of the rat increases ease of procedures, such as surgery, sampling, pharmacological development, stereotaxic neurological studies, neuroimaging and cardiovascular monitoring [1], [2]. Inbred strains of rats are often preferred due to their identical and fixed genetic background among individuals.

Mouse models generated using embryonic stem (ES) cell-based gene engineering technologies have significantly contributed to advances in biomedical research. Derivation of germline competent rat ES cells will allow the production of rat models with targeted genetic alterations using the same methods that have been so successful in the mouse [3], [4], [5]. For example, ES cell-based genetic modification has been proven to be an effective method for the production of animal models with complicated designs, such as conditional or inducible knockouts [6], [7].

Germline competent rat ES cell lines have been derived from Dark Agouti [3], [4], Sprague Dawley [3], [8], Wistar [9], and LEA [9]. Fischer344 rats are a popular strain for biomedical research in the areas including oncology, toxicology, carcinogenicity, aging and autoimmunity. However, proven germline competent ES cells lines from the F344 strain have yet to be established despite efforts by multiple laboratories worldwide [3], [4], [10].

In these studies, we describe the isolation of a novel germline competent rat ES cell line derived from Fischer344 rats carrying an EGFP transgene. We describe the characterization of ES cell lines using various prescreening tests to select rat ES cell lines that have a higher probability for germline transmissibility and the use of hybrid recipient embryos to improve the efficiency of germline competency testing.

## Materials and Methods

### Ethics Statement

This study was carried out in strict accordance with the recommendations in the Guide for the Care and Use of Laboratory Animals of the National Institutes of Health. The protocol was approved by the Animal Care and Use Committee of the University of Missouri.

### Derivation of ES Cell Lines from Transgenic Rats

Unless specifically indicated, all chemicals were obtained from Sigma-Aldrich (Sigma-Aldrich, St Louis, MO). Male F344-Tg (EGFP) F455/Rrrc (RRRC# 307) rats were obtained from the Rat Resource and Research Center (University of Missouri, www.rrrc.us) and were used as founder animals for the derivation of rat ES cell lines. This strain is homozygous for a single copy of an EGFP transgene under control of a human Ubiquitin C promoter with the woodchuck hepatitis virus post-transcriptional regulatory element (WRE) on a Fischer 344 (F344) genetic background [11]. The transgene insertion site is on Chromosome 5 (www.rrrc.us) [12]. Wild-type F344/Hsd females (Harlan, Indianapolis, IN) were mated to homozygous F344-Tg (EGFP) F455/Rrrc males. Blastocysts, all of which were hemizygous for the transgene and were positive for EGFP expression, were collected on Day 4.5 post mating in mRiECM+22 mM HEPES [13]. After collection, ES cells were isolated from blastocysts using a protocol described previously [3]. For ES cell derivation, blastocysts were treated briefly with acidic Tyrode’s solution to remove zona pellucidae and then cultured in N2B27+3 µM CHIR99021 (Axon Medchem BV, Groeningen, The Netherlands) +0.5 µM PD0325901 (Selleckchem, Houston, TX) [14] on CF-1 mouse feeder cells (Millipore, Billerica, MA) in Nunc 4-well plates (Thermo Scientific, Roskilde, Denmark) at 37°C in an incubator with 5% CO_2_ and maximal humidity. The CF-1 feeder cells were plated onto Nunc 4-well plates one day prior to the culture of embryos at a density of approximately 50,000 cells/cm2 as suggested by the manufacturer. On Day 5, outgrowths of the embryos were individually disassociated into single cell suspension using accutase and then cultured in 24-well plates. ES cells were passaged every 48–72 hours.

### ES Cell Genotyping

Selected ES cell lines were genotyped by PCR to identify their sex chromosome composition. Primers used to detect the X chromosome were 5′-GTG AAG GAG GAA TTA GGT GG-3′ and 5′-GAT GTG GTA ATT GTC ATC AC-3′ [15]. Primers used to detect the Y chromosome were 5′-GTA GGT TGT TGT CCC ATT GC-3′ and 5′-GAG AGA GGC ACA AGT TGG C-3′ [16]. PCR was performed on 20 µl reactions containing ∼10 ng genomic DNA, 1 unit of FastStart *Taq* DNA Polymerase (Roche), 750 nM of each primer, 200 µM each dNTP, and 1X Reaction buffer containing MgCl_2_ (Roche). PCR conditions were 94°C for 5 minutes, then 35 cycles of 94°C for 1 min., 61°C for 1 min. and 72°C for 1 minute followed by 72°C for 7 min. Amplicons of 272 bp (Y chromosome) and 1100 bp (X chromosome) were detected by gel electrophoresis on 1% 1X TBE agarose gels. Male ES cell lines were selected for subsequent assays.

### Expression of Pluripotency Factors

The expression of *Oct4*, *Sox2*, and *Nanog* in the established ES cell lines were examined by RT-PCR analysis using rat specific primers: *Oct4,* 5′-CCCAGCGCCGTGAAGTTG-GA-3′ and, 5′-ACCTTTCCAAAGAGAACGCCCAGG-3′; *Sox2*, 5′-ATTACCCGCAGCAAAATGAC-3′ and, 5′-AT-CGCCCGGAGTCTAGTTCT-3′; *Nanog*, 5′-GACTAGCAACGGCCTGACTCA-3′ [3] and, 5′-CTGCAATGGATGCTGGGATA-3′; *GAPDH*, ATCACTGCCACTCAGAAG-3′ and, AAGTCACAGGAGACAACC-3′ [3]. Germline-competent rat ES cell line DAc8 [7] (RRRC# 464) obtained from the Rat Resource and Research Center served as a positive control. The negative controls were rat embryonic fibroblasts (made in house), mouse embryonic fibroblasts (feeder cells, Millipore) and a no template control. RNA was extracted from up to 5×10^5^ cells using RNeasy Plus Micro Kit (QIAGEN, Valencia, CA). The High Capacity First Strand Synthesis Kit from Applied Biosystem (Carlsbad, California) was used to synthesize cDNA from 1 µg of RNA. RT-PCR was performed in 25 µl reactions containing 250 pg −250 ng cDNA, 1X PCR Buffer (Roche, Indianapolis, IN), 1.5 mM MgCl_2_, 0.2 mM dNTPs, 0.2 µM of each primer and 2.5 U of Roche FastStart *Taq* polymerase. Thermal cycling conditions were 1 cycle at 95°C, 2 min; 35 cycles of 95°C, 30 sec., 61°C, 30 sec and 72°C, 30 sec; 1 cycle at 72°C, 5 min. The DNA samples were analyzed using the QIAxcel (QIAGEN) with the QIAxcel DNA Screening Kit, QX Alignment Marker 15 bp/3 kb, and QX DNA Size Marker 100 bp-3 kb. The method was AM320 with an injection of 10 s at 5 kV and a separation of 320 s at 6 kV.

### ES Cell Karyotyping

Rat ES cells were treated with 0.1 µg/ml colcemid (Irvine Scientific, Santa Ana, CA) for 1 h at 37 °C when they reach 60–70% confluent. At the end of colcemid treatment, ES cell colonies were harvested and disassociated into single cell suspension with accutase and then pelleted by centrifugation at 200×g for 8 min in a 15 ml conical tube. After removing the supernatant, the cells were resuspended with 4–5 ml hypotonic solution (0.075 M KCl solution) and incubated at room temperature for 15 min. A few drops of freshly made fixative consisting of methanol: acetic acid (Fisher Scientific, Pittsburg, PA) in a ratio of 3∶1 were then added to the hypotonically treated cell suspension and mixed by inversion. The cells were pelleted at 200×g for 8 min and were then resuspended in 4–5 ml fixative and re-pelleted at 200×g for 8 min. After one more repetition of the fixation step, the fixed ES cells were pelleted by centrifugation at 200×g and resuspended in 1 ml fixative. Preparation of chromosome spreads and karyotype analysis of the fixed cells were performed by Dr. Chin-Lin Hsieh (Arcadia, CA). ES cell lines were analyzed by Giemsa-Trypsin-Wrights (GTW) banding and at least 20 metaphase spreads were counted. A cell line with 70% or higher metaphase spreads exhibiting a normal number of chromosomes was considered to have a normal karyotype. The passage numbers at the time of karyotyping for each cell line were passage 6 and 13 for F344-Tg(EGFP).EC4011 and passage 7 for F344-Tg(EGFP).EC4013.

### Pathogen Screening of Rat ES Cells

Both the culture media and the cell lines were subjected to pathogen screening. One milliliter of culture medium from each cell line was submitted to IDEXX-RADIL (Columbia, MO) for microbiological evaluation. The medium was placed on blood agar (BA) and Brain Heart Infusion broth (BHI) broth for 10 days to evaluate bacterial growth. Pathogen screening for ES cell lines was usually conducted after examination of the expression of pluripotent factors and karyotyping. One million cells from selected ES cell line were submitted to IDEXX-RADIL for a comprehensive pathogen testing. This included screening for the presence of H1 parvovirus, Kilham’s rat virus, *Mycoplasma* spp., rat minute virus, and rat parvovirus in the cell lines. A portion of the cell sample was also grown on BA/BHI broth for 10 days to examine any potential bacterial contamination in the cell lines.

### Chimeric Animal Production and Breeding

Male ES cell line, F344-Tg(EGFP).EC4011 was selected for the production of chimeric animals. Six days prior to blastocyst injection, cells frozen at passage 13 were thawed and cultured in N2B27+2i with CF-1 mouse feeder cells in 60 mm culture dishes and passaged every 48 h to ensure that the ES cells were fully recovered from any stress resulting from cryopreservation. On the day of injection, rat ES colonies were detached from the feeders by gently pipetting the media up and down followed by collection into a 15 ml centrifugation tube. After centrifugation at 200×g for 3 min and removing the supernatant, the pelleted ES cell colonies were disassociated with accutase into a single cell suspension followed by centrifugation at 200×g for 3 min. The cell pellet was resuspended in N2B27+20 mM HEPES and incubated on ice. Donor blastocysts were collected from Day 4.5 pregnant SD females that had been mated with DA males (Harlan). These females were synchronized using GnRH at 40 µg/rat 4 days before the mating. Donor blastocysts were cultured in mRiECM +10% fetal bovine serum (FBS) after collection. Blastocyts were injected in groups of 10, in 20 ul m-RECM-1-HEPES.ES cells were freshly added to each injection drop. Ten (10) to 12 rat ES cells were injected into single blastocysts using a beveled Transfertip (Eppendorf, Hauppauge, NY). After injection of each group, injected blastocysts were immediately transferred into mRiECM +10% FBS and cultured for about 1 hour. Approximately 20–30 blastocysts were transferred into the uterine horns of Day 3.5 pseudo-pregnant SD females (10–15 blastocysts per uterine horn). All surgical procedures were approved by the Animal Care and Use Committee of the University of Missouri-Columbia.

Chimeric animals were identified from the resulting pups by coat color chimerism (presence of albino hairs against an agouti coat color background). Upon sexual maturation, chimeric animals were bred with SD mates to verify germline transmissibility. The inheritance of ES cell genetics in the offspring was assessed by the presence of the EGFP transgene using an insertion site specific PCR genotyping assay developed by RRRC (www.rrrc.us). DNA was extracted from tail biopsies using the Extract-N-Amp Tissue PCR kit and PCR was performed using the manufacturer’s protocol and reagents. Primers are LWS 455 5F: 5′-AAC CTC CCA GTG CTT TGA ACG CTA-3′; LWS 455 5R: 5′-GGT GCC AAG CCT CAA CTT CTT TGT-3′ and U3r-4 5′-ATC AGG GAA GTA GCC TTG TGT GTG-3′. Thermal cycling conditions were 1 cycle at 94°C, 3 min; 35 cycles of 94°C, 30 sec., 64°C, 30 sec and 72°C, 1 min; 1 cycle at 72°C, 10 min. The wild type product is 438 bp and the mutant product is 129 bp. Recovery of animals that inherited the transgene from their chimeric parent was evidence of germline competency of the ES cell line. Failure to produce any transgenic offspring in three consecutive litters was taken as lack of germline competency.

## Results

### Derivation of ES Cell Lines from F344-Tg (EGFP) F455/Rrrc Transgenic Rats

A total of 34 blastocysts were collected from 12 F344 females mated with F344-Tg (EGFP) F455/Rrrc males. After removal of zona pellucidae, these 34 blastocysts were successfully cultured and showed outgrowths. After several passages, a total of 27 ES cell lines were established from the 34 blastocysts.

### Characterization of the Novel ES Cell Lines

**Figure 1:**
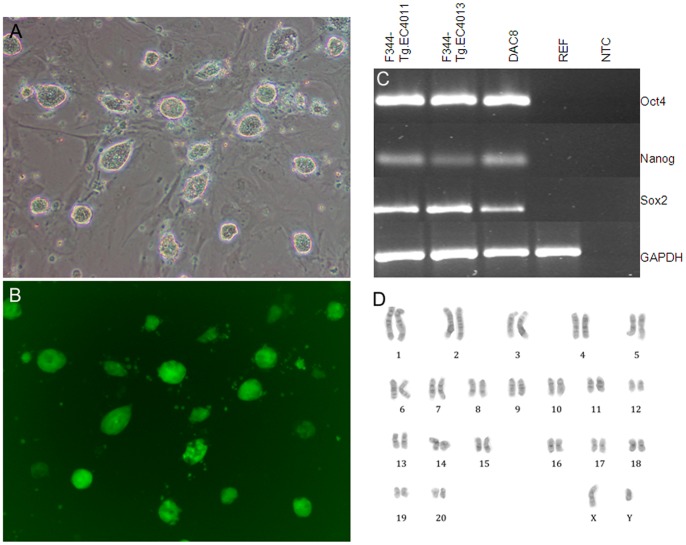
ES cell morphology and karyotype. The morphology and karyotype of F344-Tg.EC4011 is shown and is representative of the other ES cell lines. (A) Phase contrast image shows cultured ES cells forming compact colonies with smooth edges. (B) Fluorescence microscopy image of same field of view as (A). Cultured ES cells express the EGFP transgene. Scale bar represents 100 µm. (C) RT-PCR analysis of *Oct4*, *Nanog*, and *Sox2* gene expression using rat specific primers. DAc8, a proven germline competent rat ES cell line (Li et al., 2008) is included as a positive control; rat embryonic fibroblasts (REFs), mouse embryonic fibroblasts (MEFs) as well as a no template control (NTC) are also shown. (D) Cytogenetic analysis. ES cells have a normal male karyotype (42, XY).

All 27 cell lines could be maintained in an undifferentiated state in rat ES medium (N2B27+2i) and showed compact colonies with smooth boundaries and retained GFP fluorescence (Fig. 1A and 1B). Of these 27 cell lines, 14 were chosen randomly for further analysis. Genotyping results for the fourteen lines (F344-Tg.EC4001 to F344-Tg.EC4014) showed that F344-Tg.EC4011 and F344-Tg.EC4013 are male cell lines and the other 12 lines were female lines. The two male lines expressed pluripotency factors *Oct4*, *Sox2*, and *Nanog* by RT-PCR analysis and had normal karyotypes at Passage 6 and 7, respectively (Fig. 1C and 1D). F344-Tg.4011 was karyotyped again at Passage 13 with 15/20 cells examined exhibiting a normal karyotype. Pathogen screening indicated that the two male lines (F344-Tg.EC4011 and F344-Tg.EC4013) were free of H1 parvovirus, Kilham’s rat virus, *Mycoplasma* spp., rat minute virus, and rat parvovirus. There was also no bacterial or fungal growth after 10 days of sterility testing for both the culture media as well as the cell lines.

### Generation of Chimeras

**Figure 2:**
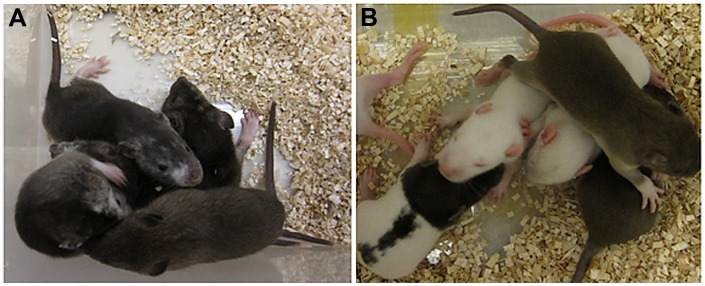
Coat color chimeras and their offspring. (A) Chimeric animals (albino patches on face) from F344-Tg.EC4011 ES cell injections into SD X DA blastocysts. (B) Offspring from chimeric animal breeding.

Based on karyotyping results, ES cell line F344-Tg.EC4011 had a higher percentage of cells with a normal male karyotype than line F344-Tg.EC4013, and therefore this line was chosen for further analysis. Ten to twelve F344-Tg.EC4011 ES cells at passage 16 were injected into hybrid DA X SD blastocysts. A total of 199 blastocysts were injected and transferred. Seventy-five live pups were produced. A total of 13 animals (11 males and 2 females) showed coat color chimerism (Table 1 and Figure 2A).

**Table 1:** Production of chimeric animals via blastocyst injection with rat ES cell line F344-Tg.EC4011.

Blastocystinjected	ES cells(passage #)	Embryos		Total pups	Chimericanimals (sex)
		injected	transferred		
1^st^ injection	F344-Tg.EC4011(P16)	55	55	24	8 (M)
2^nd^ injection	F344-Tg.EC4011(P16)	39	39	17	2 (1 M and 1 F)
3^rd^ injection	F344-Tg.EC4011(P16)	61	61	17	2 (M)
4^th^ injection	F344-Tg.EC4011(P16)	44	44	17	1 (F)

### Demonstration of Germline Competency

Eleven male chimeric animals derived from the F344-Tg.EC4011 cell line were bred to SD mates. Once a chimeric animal produced a GFP positive pup, indicating its ability to pass on the transgene derived from the ES cells, breeding was stopped. Breeding was also discontinued after a chimeric animal failed to produce a GFP positive pup within three litters. One animal (468RII) did not produce any offspring after the second litter. The results showed that 6 out of the 11 chimeric animals derived from cell line F344-Tg.EC4011 were able to transmit the ES cell-derived EGFP gene through the germline (Table 2 and Figure 2B).

**Table 2:** Breeding results of chimeric animals derived from rat ES cell line F344-Tg.EC4011.

Chimeric animals	1^st^ litter		2^nd^ litter		3^rd^ litter		Germline competence
	Total	GFP+	Total	GFP+	Total	GFP+	
465RII	11	7					+
466RII	8	2					+
467RII	13	1					+
468RII	2	0	10	0			–
469RII	4	0	3	0	5	0	–
470RII	7	1					+
471RII	13	1					+
472RII	12	0	8	1			+
817RII	1	0	17	0	6	0	–
913RII	14	0	11	0	13	0	–
914RII	16	1					+

## Discussion

In the present study, we report derivation of a novel rat ES cell line with germline transmissibility from transgenic F344 rats carrying a ubiquitously expressed EGFP gene on Chromosome 5. We also demonstrate that hybrid recipient embryos with a SD × DA genetic background were able to support the germline competence testing of the F344-Tg.EC4011 embryonic stem cell line.

The establishment of germline competent ES cell lines from inbred rats will provide valuable resources for the creation of new genetically engineered rat models on inbred genetic backgrounds. It has been shown in mice that variations in genetic background can have a profound influence on the phenotypes of genetically altered animals [17]. Therefore, inbred animals are usually the preferred animals for the generation of mutant animal models [18]. F344 rats are a popular strain used in both biomedical and drug discovery. However, similar to mice, the derivation of germline competent ES cell lines in rats is also highly strain-dependent [3], [4], [19], [20]. ES cell lines from F344 genetic background have been generated in at least three laboratories. None of these ES cell line has been demonstrated to be able to transmit through the germline. In fact, the existing putative F344 ES cell lines even failed to give rise to chimeric animals [3], [4], [20].

The genetic combination of ES cells and the host embryos is a key factor governing the ES cells’ ability to colonize the gonads. There are several factors that have been demonstrated to affect the ES cell’s ability to transmit their genetic material through the germline including the genetic background, stemness, normality of karyotype, pathogen status of the ES cell line as well as the genetic background of recipient embryos [7], [21]. Among these factors, the combination of the genetic background of the ES cells and the recipient embryos has been demonstrated to be a critical factor affecting the germline transmissibility of the ES cells [7], [21], [22]. Ideally, the host background should allow the ES cells to have an optimal developmental advantage when injected into the blastocyst. This allows the ES cells to contribute to the germline of the chimeric animals which consequently transmit the ES cell-derived genetic material to their offspring [3], [7], [19]. Because a relatively few number of ES cell lines have been isolated to date and even fewer have been shown to be germline competent, relatively little is known about the optimal combinations of ES cell genetic background and recipient blastocyst genetic background in the rat. ES cell lines from F344 genetic background have been injected into host blastocysts from DA rats [3], F344 [20] or SD rats [20]. These ES cell lines failed to generate chimeric animals. Similarly, recipient embryos from SD rats have been used as recipient embryos for DA ES cell lines to generate chimeric animals, however, these chimeric animals failed to produce offspring with an ES cell genetic contribution [3]. Chimeric animals resulting from SD blastocysts injected with ES cells from Brown Norway rats also failed to produce offspring with the Brown Norway genetic background [19].

In our study, we successfully used DA x SD hybrid blastocysts as recipient embryos. SD female rats were selected because of their high fecundity while male DA rats were selected for their pigmented coat color to aid in detection of chimeric animals. Chimeric animals were generated through blastocyst injection of ES cells from F344-Tg.EC4011 and the ES cells were able to colonize the gonads and produce sperm as evidenced by the transmission of the ES cell-derived EGFP gene from the chimeras to their offspring.

In conclusion, novel germline competent F344 rat ES cell line is now available and this particular ES cell line has the added advantage that it carries an EGFP transgene. The cell line has been deposited in the Rat Resource and Research Center (RRRC), assigned stock number RRRC#654, and is available for distribution to the research community. This fluorescently-tagged F344 ES cell line provides an extremely useful tool for investigators who want to make genetically engineered rat models directly in a F344 genetic background.
